# The interaction effects between depression and sleep status on asthma: a national cross-sectional study

**DOI:** 10.3389/fpsyt.2024.1487550

**Published:** 2024-10-16

**Authors:** Yuxin Lai, Xiaomei Zhang, Huan Dong, Mengqian Li

**Affiliations:** ^1^ Department of Internal Medicine of Chinese Medicine, Beijing University of Chinese Medicine, Beijing, China; ^2^ Department of Ming Yi Tang Pulmonary Nodule and Chest Disease Center, Dongfang Hospital Beijing University of Chinese Medicine, Beijing, China; ^3^ Department of Respiratory Medicine, Changping Hospital of Integrated Chines and Western Medicine, Beijing, China

**Keywords:** asthma, depression, sleep disorders, sleep duration, NHANES

## Abstract

**Background:**

Asthma, depression, and sleep problems are three significant public health issues that are closely interrelated. This study aims to explore the relationship between depression, sleep status and asthma, as well as the potential interaction among these conditions and their effects on asthma.

**Method:**

This cross-sectional study utilized data from the 2005-2008 National Health and Nutritional Examination Survey, including information on asthma, depression, sleep status and confounding factors. Multivariate logistic regression analyses were conducted to investigate the relationship between depression, sleep status, and asthma. Subgroup analyses were conducted to test the p-interaction between depression and each stratified variable. Additionally, both multiplicative and additive approaches were employed to assess the interaction between depression and sleep status on asthma, as well as to quantify their combined effects.

**Results:**

A total of 8,327 participants (mean age 46.53 years) were included in this study. Compared to the individuals without depression, those with depression have an increased risk of asthma [Odds ratio (OR) = 1.57, 95% Confidence interval (CI) = 1.22-2.03], and an increase in the severity of depressive symptoms is associated with a higher risk of developing asthma. Additionally, poor sleep quality, sleep disorders, and insufficient sleep was associated with an increased risk of asthma. Effect modification was observed between depression and PIR status, smoking status, and sleep disorders in relation to asthma (p-interaction <0.05). Moreover, we found a positive interaction between severe depression and excessive sleep (OR = 29.07, 95% CI = 3.24-260.38). Furthermore, we observed the quantitative additive interaction indicators between moderately severe depression and insufficient sleep [Relative excess risk due to interaction (RERI) = 1.63, 95%CI = 0.18-3.83; Attributable proportion (AP) = 0.51, 95%CI = 0.15-0.87; Synergy index (SI) = 3.92, 95%CI = 1.65-23.50] influencing asthma risk.

**Conclusion:**

Our study revealed distinct associations between depression, the severity of depressive symptoms, poor sleep quality, sleep disorders, and insufficient sleep with asthma. Additionally, there was an interaction between moderately severe depression and insufficient sleep on asthma. Psychological and sleep assessment are essential in asthma management. Clinicians should consider the potential risk of depression and sleep problems in asthma patients and intervene. Further longitudinal research is needed to better understand the pathophysiological mechanisms behind the interactions between asthma, depression, and sleep problems.

## Introduction

Asthma is a common chronic respiratory disease that poses a significant public health challenge worldwide (1, 2). It is estimated that up to 300 million individuals are affected by asthma (3). Its growing impact on governments, healthcare systems, families, and individuals is becoming increasingly substantial globally (4, 5). Furthermore, a considerable proportion of individuals with asthma also experience co-morbid depression and sleep problems (6, 7).

The association between asthma and depression is bidirectional (8, 9). Individuals with asthma have higher odds of developing depression compared to individuals without asthma (8). Additionally, depression is a well-established risk factor for the onset and progression of asthma, as well as for impacting asthma control and even affecting the death of individuals with asthma (10, 11).

The relationship between sleep and asthma is complex. Asthma is associated with lower subjective sleep quality and sleep disturbances (12). Individuals with asthma exhibit increased sleep latency, reduced sleep quality and efficiency, which can worsen as asthma severity increases (12, 13). Besides, the presence of sleep problems, including sleep disorders, abnormal sleep duration, insomnia, and obstructive sleep apnea (OSA), among others, has been associated to increase the risk of asthma (13–15). Sleep disorders can impact various health factors, including cognitive function, asthma control, and quality of life (16, 17).

Consequently, shared pathways may exist between depression and sleep problems, which mutually influence asthma. The existence of this interactive effect may suggest that the risk of asthma in individuals with depression could be further increased by the presence of sleep problems. However, prior investigations have focused on the association between sleep problems and depression on asthma, the interaction between these conditions has not been well described (18–20). Therefore, by gaining a better understanding of the associations among these conditions, we can enhance asthma management by effectively addressing underlying risk factors, improving the quality of life for individuals with asthma, and reducing the global burden. This study aimed to explore the relationship between depression (including its severity) and sleep status (sleep duration, sleep quality, and sleep disorders) and on asthma. To achieve this, we utilized a nationally representative sample from the National Health and Nutrition Examination Survey (NHANES).

## Method

### Sample

The data utilized in this study were obtained from the 2005-2008 NHANES, a program of the Centers for Disease Control and Prevention (CDC) aimed at compiling health and vital statistics. The survey was designed to evaluate the health and nutritional status of the US general population and employed a complex multistage probability sampling design that oversampled minorities and older adults to enhance generalizability (21). During each 2-year survey, participants were interviewed in their homes and subsequently visited a mobile examination center, where they completed additional questionnaires, underwent physical examinations, and provided blood samples. A standardized questionnaire was used to gather data on participants’ age, race and ethnicity, gender, educational background, income and medical history (22). The survey was conducted in accordance with the ethical standards and guidelines established by the National Center for Health Statistics (NCHS), and written informed consent was obtained from all adult participants prior to data collection.

**Figure 1:**
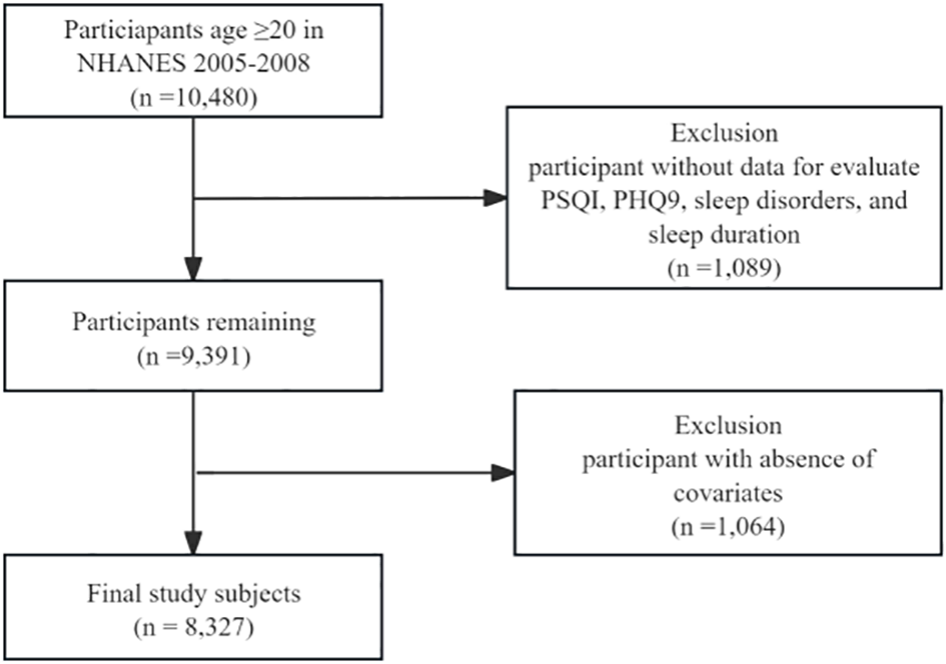
Flowchart of the study design and participants. NHANES, National Health and Nutrition Examination Survey; PSQI, Pittsburgh Sleep Quality Index; PHQ-9, Patient Health Questionnaire-9.

In this cross-sectional study, we analyzed data from a total of 8,327 adults between the ages of 20 and 80 who participated in the NHANES study from 2005-2008, covering two survey cycles. Exclusion criteria established by the study design included the following (participants with partial missing data were entirely excluded): [1] age < 20 years; [2] missing data for evaluate the Pittsburgh Sleep Quality Index(PSQI), sleep disorders, and sleep duration; [3] missing data for Patient Health Questionnaire-9 (PHQ-9); and [4] respondents with incomplete main covariates. The selection process is detailed in **Figure 1**.

### Assessment of asthma

In light of the potential influence of bronchodilators and anti-asthma medications on outcomes, we employed medication usage as a screening criterion for asthma. Data were retrieved from the NHANES database utilizing the keywords “bronchodilator/anti-asthma medication,” encompassing selective phosphodiesterase-4 inhibitors, mast cell stabilizers, leukotriene modifiers, and inhaled corticosteroids. Asthma was evaluated based on the following criteria (20): [1] self-reported diagnosis of asthma by a healthcare professional, [2] use of anti-asthmatic medications, and [3] use of bronchodilator/anti-asthma medication under the age of 40 who are non-smokers and do not have chronic bronchitis or emphysema.

### Assessment of depression

Depression was evaluated using the Patient Health Questionnaire-9 (PHQ-9), which comprises the 9-item screening instrument was used to inquire about the frequency of depressive symptoms experienced in the preceding two weeks. Severity of depressive symptoms was categorized as follows: minimal depression (PHQ-9 score 1–4), mild depression (PHQ-9 score 5–9), moderate depression (PHQ-9 score 10–14), moderately severe depression (PHQ-9 score 15–19), and severe depression (PHQ-9 score 20–27). A total score ≥ 10 was adopted to determine clinically relevant depression (CRD) according to the fourth edition of the Diagnostic and statistical Manual of Mental Disorders (DSM-IV) (23, 24).

### Assessment of sleep status

The Pittsburgh Sleep Quality Index (PSQI) was developed to access the quality and patterns of sleep in both adolescents and adults (25). In the NHANES 2005-2008, based on three indicators: sleep latency, sleep disturbance and daytime dysfunction, eight self-reported items were used to assess the Pittsburgh Sleep Quality Index (PSQI). Sleep latency was assessed using two items: (1) “How long does it usually take you to fall asleep at bedtime (minutes)?” and (2) “In the past month, how often did you have trouble falling asleep?”. Sleep disturbances were evaluated using two items: (1) “In the past month, how often did you wake up during the night and have trouble getting back to sleep?” and (2) “In the past month, how often did you wake up early in the morning and was unable to get back to sleep?”. Daytime dysfunction was measured using two items: (1) “In the past month, how often did you feel unrested during the day, no matter how many hours of sleep you had?” and (2) “In the past month, how often did you feel excessively or overly sleepy during the day?”. The assigned score for the response to the frequency question mentioned above is presented as follows: 0 (never-0 time a month), 1(rarely-1 time a month), 2 (sometimes-2 to 4 times a month), 3 (often-5 to 15 times a month), and 4(almost always-16 to 30 times a month) (26). And time to fall asleep was categorized to reflect the scores as follows: 0 (≤15 minutes), 1 (16–30 minutes), 2 (31–60 minutes), and 3 (≥60 minutes) (25). The score of PSQI was calculated by adding scores of sleep latency, sleep disturbances, and daytime dysfunction (rang: 0-23). A score exceeding 5 is designated as indicative of poor sleep quality, whereby higher scores correspond to a deteriorating sleep quality (26).

Sleep disorder was assessed according to the question “Have you ever been told by a doctor or health professional that you have a sleep disorder?” (yea/no). If participants answered positively, they were identified as having a sleep disorder.

Sleep duration was self-reported by the question “How much sleep do you usually get at night on weekdays or workdays?” And the duration of sleep was classified into three categories from the recommendation of US National Sleep Foundation (27): Insufficient sleep (≤6h per night), adequate sleep (7-8h per night), excessive sleep (≥9h per night).

### Covariate assessment

To reduce potential bias, we incorporated demographic factors such as: age, sex (male, female), race (non-Hispanic white, non-Hispanic black, Mexican American, and other), educational attainment [completion of ≤12th grade, completion of high school or equivalent, completion of some college or an Associate in Arts degree (GED/AA), and completion of college or higher education)], marital status (unmarried, married or living with a partner, divorced or separated, and widowed), Poverty-to-income ratio (PIR) levels [low-income level (PIR <1), middle-income level (PIR 1-4), and high-income level (PIR >4)] (28), the body mass index (BMI) [normal weight (BMI 18.5-25.0 kg/m²), overweight (BMI 25.0-29.9 kg/m²), obese (BMI ≥30.0kg/m²)] (29, 30). Smoking status was classified into 3 categories: Never (smoked fewer than 100 cigarettes in their lifetime), Former (smoked at least 100 cigarettes in their lifetime but were not currently smoking), and Current (smoked at least 100 cigarettes in their lifetime and were currently smoking either some days or every day). Hypertension was defined as described previously (31): a mean systolic blood pressure ≥130mmHg or a diastolic blood pressure ≥80 mmHg; self-reported use of antihypertensive medication; or a self-reported diagnosis of hypertension. Any of these three criteria identified as an individual as having hypertension. Diabetes was diagnosed based on self-reported doctor-diagnosed diabetes; use of diabetic medication or insulin; glycosylated hemoglobin (HbA1c) ≥6.5%; fasting glucose ≥7.0mmol/L; random blood glucose ≥11.1mmol/L, 2-hour OGTT blood glucose ≥11.1mmol/L.

### Statistical analysis

In this study, all analyses were conducted using sample weights to produce precise nationwide estimates in the US. To mitigate possible bias, we included demographic factors (age, sex, race, education, marital status, PIR, and smoking status) and other health variables (BMI, hypertension, and diabetes). Descriptive statistics for measurement data were presented as mean and standard error (S.E.). Bivariate analyses of continuous and binary variables were performed using t-tests and Wald chi-square tests, respectively. Relative risk ratios of depression and sleep status to asthma was assessed using weighted multiple logistic regression models. Through stepwise adjustment for various risk factors, we examined different models. The model 1 represented the original unadjusted model without considering any potential confounders. Model 2 was adjusted for age, sex, race, education, marital status, smoking status, hypertension, diabetes, BMI, and PIR. Adjusted odds ratios (ORs) were calculated based on these data, using 95% confidence intervals (CIs). Subgroup and stratified analyses were conducted by age (age 20-64 years or ≥65 years), sex, race, BMI status, PIR status, smoking status, sleep quality, sleep disorders, and sleep duration. The p-interaction between depression and each stratified variable was also tested. In addition, we investigate whether there is a multiplicative interaction between sleep status and depression regarding asthma risk by examining their product. This evaluation aims to determine the nature of this interaction, whether positive or negative. To further quantify their interaction in terms of asthma risk, we employed the relative excess risk due to interaction (RERI), the attributable proportion of interaction (AP), and the synergy index (SI). RERI quantifies the excess risk attributable to the interaction between sleep status and depression, while AP indicates the proportion of the combined risk due to this interaction. SI represents the increase in risk resulting from the combined impact of both factors. If the confidence interval for RERI and AP include 0, and the confidence interval for SI includes 1, this indicates no additive interaction. All statistical analyses were carried out using R version 4.2.1 (R Foundation for Statistical Computing, Vienna, Austria). A 2-sided P value of less than 0.05 was considered statistically significant. To correct for multiple testing, 2-side P values were adjusted according to the method of Benjamin-Hochberg to control the false discovery rate (FDR) (32). Given the large sample size and covariates, there is a potential risk of overfitting of the model.

## Results

The final study included a total of 8,327 participants (weighted n=177,948,849) from NHANES 2005-2008, and 1,103 (13.25) had asthma. The main characteristics of the analyzed participants are presented in **Table 1**. For all participants, the mean (SD) age was 46.53(0.45) years. Among the participants, 49.2% were male and 50.8% were female, with the majority being non-Hispanic white (72.51%). Compared to individuals without asthma, those with asthma exhibited significant differences in sleep duration, PSQI scores, sleep quality, depression, and depressive symptom severity (*P <*0.0001). In addition, there were differences in age, sex, race, marital status, PIR status, BMI status between participants with and without asthma groups (*P <*0.05).

**Table 1:** Characteristic of all study participants.

Variables	Total (n=8,327,Weighted%)	Asthma	No asthma	P value	
		N = 1,103	N = 7,224		
**Age, years(S.E)**	46.53(0.44)	44.05(0.64)	46.94(0.45)	<0.0001	
**Age group, n(%)**				<0.001	
20-64	6310(83.87)	886(87.41)	5424(83.29)		
≥65	2017(16.13)	217(12.59)	1800(16.71)		
**Sex, n(%)**				0.001	
Female	4098(50.80)	609(56.45)	3489(49.87)		
Male	4229(49.20)	494(43.55)	3735(50.13)		
**Race, n(%)**				< 0.0001	
Non-Hispanic white	4170(72.51)	103(48.68)	3572(72.13)		
Non-Hispanic black	1793(10.82)	179(32.66)	1524(10.64)		
Mexican American	1472(7.68)	24(3.90)	1375(8.39)		
Other	892(8.99)	71(14.76)	753(8.84)		
**Marital status**, n(%)				0.001	
Married/With partner	5118(65.35)	610(60.58)	4508(66.13)		
Never married	1312(16.02)	221(20.20)	1091(15.34)		
Divorced/Separated	1208(13.05)	196(15.12)	1012(12.71)		
Widowed	689(5.59)	76(4.10)	613(5.83)		
**Education, n(%)**				0.06	
High school/GED/AA	4318(55.53)	3697(54.83)	621(59.80)		
College and above	1677(26.44)	1471(26.90)	206(23.63)		
≤12th grade	2332(18.03)	2056(18.27)	276(16.57)		
**PIR status, n(%)**				0.003	
< 1	1526(11.98)	249(15.53)	1277(11.40)		
1 – 4	4547(50.15)	572(46.97)	3975(50.67)		
> 4	2254(37.87)	282(37.49)	1972(37.94)		
**BMI status, n(%)**				< 0.0001	
Under & health weight	2437(31.96)	269(26.61)	2168(32.84)		
Overweight	2881(33.70)	322(29.75)	2559(34.35)		
Obese	3009(34.33)	512(43.63)	2497(32.81)		
**Smoking status,** n(%)				0.1	
Never	4275(51.31)	522(49.45)	3753(51.61)		
Former	2148(24.95)	284(23.90)	1864(25.12)		
Current	1904(23.74)	297(26.65)	1607(23.26)		
**Diabetes, n(%)**				0.57	
DM	1427(12.19)	220(13.75)	1207(11.94)		
No	6900(87.81)	883(86.25)	6017(88.06)		
Variables	Total (n=8,327,Weighted%)	Asthma	No asthma		P value
		N = 1,103	N = 7,224		
**Hypertension, n(%)**					0.37
Yes	4808(63.42)	585(61.65)	4223(63.71)		
No	3519(36.58)	518(38.35)	3001(36.29)		
**Sleep duration, n(S.E)**	6.86(0.03)	6.59(0.06)	6.90(0.03)		< 0.0001
**Sleep duration level, n(%)**					< 0.0001
Insufficient sleep (≤6h)	3272(36.57)	540(45.86)	2732(35.05)		
Adequate sleep (7–8h)	4458(56.76)	477(47.08)	3981(58.35)		
Excessive sleep (≥9h)	597(6.67)	86(7.06)	511(6.60)		
**PSQI score, n(S.E)**	7.59(0.07)	9.10(0.21)	7.34(0.07)		< 0.0001
**Sleep quality, n(%)**					< 0.0001
Good	3521(38.98)	337(29.26)	3184(40.57)		
Poor	4806(61.02)	766(70.74)	4040(59.43)		
**Sleep disorders, n(%)**					< 0.0001
No	7697(92.45)	947(86.93)	6750(93.36)		< 0.0001
Yes	630(7.55)	156(13.07)	474(6.64)		
**CRD, n(S.E)**					< 0.0001
No	7657(93.28)	950(89.39)	6707(93.92)		
Yes	670(6.72)	153(10.61)	517(6.08)		
**Depressive symptom severity**					< 0.0001
Minimal depression	6425(78.66)	726(69.55)	5699(80.16)		
Mild depression	1232(14.62)	224(19.84)	1008(13.77)		
Moderate depression	437(4.44)	96(6.93)	341(4.04)		
Moderately severe depression	172(1.70)	38(2.37)	134(1.60)		
Severe depression	61(0.57)	19(1.32)	42(0.45)		

AA, Associate of Arts; GED, General Educational Development; BMI, body mass index; PIR, Poverty-to-income ratio; PSQI, Pittsburgh Sleep Quality Index; DM, diabetes mellitus; IFG, impaired fasting glucose; IGT, Impaired glucose tolerance; PSQI, Pittsburgh Sleep Quality Index; CRD, clinically relevant depression.

### Association between sleep status and asthma

The association between sleep status (sleep duration, sleep quality, and sleep disorders) and asthma are shown in **Table 2**. After adjusted for covariates, the association between sleep status (sleep duration, sleep quality, and sleep disorders) and asthma remained stable. Compared to individuals with adequate sleep (7-8h), those with insufficient sleep (≤6h) had a higher risk of asthma in both Model 1 (OR = 1.62, 95% CI: 1.40-1.88, *P <*0.0001) and Model 2 (OR = 1.52, 95% CI: 1.31-1.77, *P <*0.0001). However, there was no statistically significant connection between the excessive sleep (≥9h) and asthma in either Model 1 (OR = 1.33, 95% CI: 0.96-1.83, *P* = 0.08) or Model 2 (OR = 1.62, 95% CI: 0.87-1.82, *P* = 0.19). In comparison to good sleep quality, participants with poor sleep quality were associated with a higher risk of asthma in both Model 1 (OR = 1.65, 95% CI: 1.32-2.06, *P <*0.0001) and Model 2 (OR = 1.50, 95% CI: 1.18-1.92, *P* = 0.003). Additionally, we examined the relationship between sleep disorders and asthma. Individuals with sleep disorders had a higher risk of asthma compared to those without sleep disorders in both Model 1 (OR = 2.11, 95% CI: 1.65-2.70, *P <*0.0001) and Model 2 (OR = 1.92, 95% CI: 1.47-2.52, *P <*0.001).

**Table 2:** Multivariate logistic regression of sleep pattern on asthma.

Variables	Model 1 OR(95% CI)	P value	P value*	Model 2 OR(95% CI)	P value	P value*
Sleep duration						
Adequate sleep	Ref			Ref		
Insufficient sleep	1.62(1.40,1.88)	<0.0001	<0.0001	1.52(1.31,1.77)	<0.0001	<0.0001
Excessive sleep	1.33(0.96,1.83)	0.08	0.08	1.26 (0.87,1.82)	0.19	0.19
Sleep quality						
Good	Ref			Ref		
Poor	1.65(1.32,2.06)	<0.0001	<0.0001	1.50(1.18,1.92)	0.003	0.004
Sleep disorders						
No	Ref			Ref		
Yes	2.11(1.65,2.70)	<0.0001	<0.0001	1.92(1.47,2.52)	<0.001	<0.001

(Ref, reference; OR, odds ratio; CI, confidence interval).

*Adjusted P values were corrected via the false discovery rate by using the Benjamin-Hochberg method.

Model1: unadjusted model.

Model2: adjustment for age, sex, race, marital status, education, smoking status, hypertension, diabetes, BMI, PIR.

### Association between depression and depressive symptoms severity on asthma

Our investigation focused on the impact of depression on asthma through using multiple models, as shown in **Table 3**. After adjusted for covariates, the association between depression, its severity, and asthma remained stable. Depression was associated with a higher risk of asthma in both Model 1 (OR = 1.82, 95% CI: 1.45-2.29, *P <*0.0001) and Model 2 (OR = 1.57, 95% CI: 1.22-2.03, *P* = 0.002). Furthermore, our study explored the relationship between the severity of depressive symptoms and asthma. Individuals with more severe depressive symptoms exhibited a significantly higher risk of asthma in both Model 1 and Model 2, except for those with moderate severe depression. These findings indicate that an increase in the severity of depressive symptoms is associated with a higher risk of developing asthma.

**Table 3:** Multivariate logistic regression of depression and depressive symptoms severity on asthma.

Variables	Model 1 OR(95% CI)	P value	P value*	Model 2 OR(95% CI)	P value	P value*
Depression						
No	Ref			Ref		
Yes	1.82(1.45,2.29)	<0.0001	<0.0001	1.57(1.22,2.03)	<0.01	0.01
Depressive symptom severity						
Minimal depression	Ref			Ref		
Mild depression	1.66(1.33,2.08)	<0.0001	<0.0001	1.52(1.16,1.98)	0.006	0.015
Moderate depression	1.98(1.47,2.66)	<0.0001	<0.0001	1.70(1.20,2.39)	0.007	0.012
Moderately severe depression	1.71(1.07,2.72)	0.03	0.03	1.48(1.02,2.51)	0.04	0.04
Severe depression	3.40(1.69,6.85)	0.001	0.001	2.61(1.17,5.82)	0.02	0.03

(Ref, reference; OR, odds ratio; CI, confidence interval).

*Adjusted P values were corrected via the false discovery rate by using the Benjamin-Hochberg method.

Model1: unadjusted model.

Model2: adjustment for age, sex, race, marital status, education, smoking status, hypertension, diabetes, BMI, PIR.

### Subgroup analysis

**Figure 2:**
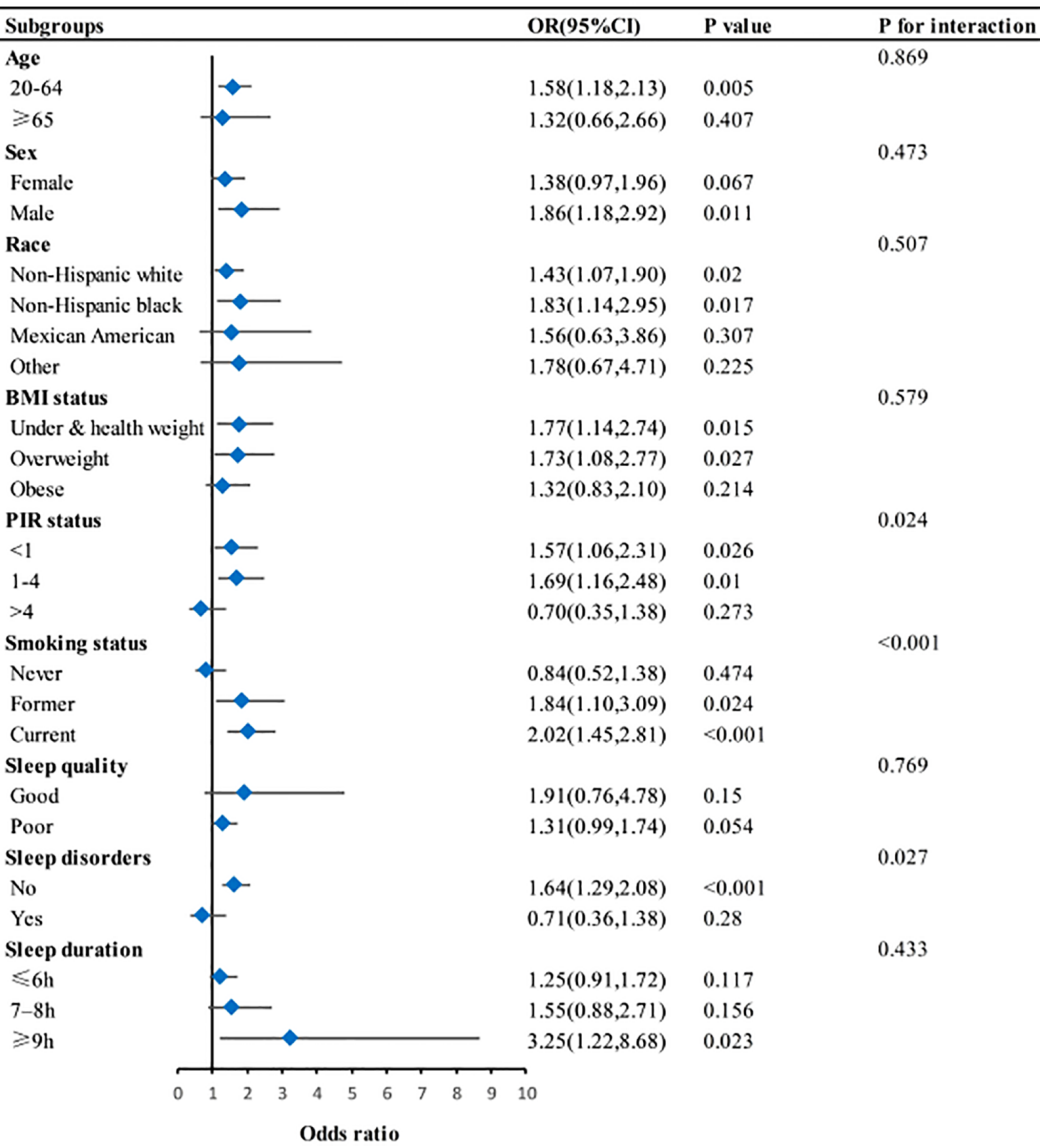
Subgroup analysis for association between depression and asthma. BMI, body mass index; PIR, Poverty-to-income ratio. Adjusted for age, sex, race, marital status, education, smoking status, hypertension, diabetes, BMI, and PIR. Except for subgroup variable.

**Figure 2** presents the results of subgroup and stratified analyses. Effect modification was discovered between depression and PIR status, smoking status, and sleep disorders in relation to asthma (*P* for interaction <0.05). The effect size of depression on asthma remained stable across the other subgroups.

### Interaction between depression and sleep status on asthma

We assessed the multiple interactions between sleep status (insufficient sleep, excessive sleep, poor sleep quality, and sleep disorders) and depression on asthma, as present in **Table 4**. However, we found no statistically significant contribution of the product of sleep status (insufficient sleep, excessive sleep, poor sleep quality, and sleep disorders) and depression to asthma risk (all *P* ≥0.05).

**Table 4:** Multiplicative interaction between depression and sleep status on asthma.

Variables	Model 1 OR(95% CI)	P value	P value*	Model 2 OR(95% CI)	P value	P value*
Subgroup 1	0.92(0.39,2.19)	0.85	0.85	0.89(0.35,2.27)	0.78	0.78
Subgroup 2	0.59(0.32,1.08)	0.08	0.32	0.53(0.28,1.01)	0.05	0.20
Subgroup 3	0.91(0.52,1.58)	0.73	0.97	0.92(0.49,1.74)	0.77	1.00
Subgroup 4	1.47(0.57,3.81)	0.41	0.82	1.62(0.55,4.78)	0.34	0.68

[Subgroup 1: Depression*Poor sleep quality; Subgroup 2: Depression*Sleep disorders; Subgroup 3: Depression*Insufficient sleep (≤6h); Subgroup 4: Depression* Excessive sleep (≥9h)].

(Ref, reference; OR, odds ratio; CI, confidence interval).

*Adjusted P values were corrected via the false discovery rate by using the Benjamin-Hochberg method.

Model1: unadjusted model.

Model2: adjustment for age, sex, race, marital status, education, smoking status, hypertension, diabetes, BMI, PIR.

### Interaction between depressive symptoms severity and sleep status on asthma

To further assessed the multiple interactions, we evaluated the effects of the severity of depressive symptoms (moderate depression, moderately severe depression, and severe depression) and sleep status (insufficient sleep, excessive sleep, poor sleep quality, and sleep disorders) on asthma, as shown in **Table 5**. After incorporating depressive symptoms severity and sleep status into a multivariate logistic regression model, we observed a statistically significant contribution of the product of moderately severe depression and insufficient sleep (OR = 3.28, 95% CI: 1.20-8.9, *P* = 0.02), as well as from severe depression and excessive sleep (OR = 29.07, 95% CI: 3.24-260.38, *P <*0.001). These finding indicate a potential positive synergistic effect between moderately severe depression and insufficient sleep, as well as between severe depression and excessive sleep. However, the synergistic effect between moderately severe depression and insufficient sleep became no statistically significant after the Benjamin-Hochberg correction (*P*-adjust = 0.12).

**Table 5:** Multiplicative interaction between depressive symptoms severity and sleep patterns with asthma.

Variables	Model 1 OR(95% CI)	P value	P Value*	Model 2 OR(95% CI)	P value	P value*
Depression severity 1 * Sleep pattern 1	0.86(0.28, 2.58)	0.77	0.84	0.83(0.20, 3.55)	0.74	0.74
Depression severity 1 * Sleep pattern 2	0.54(0.26,1.13)	0.54	1.00	0.50(0.19,1.32)	0.12	0.36
Depression severity 1 * Sleep pattern 3	0.64(0.35, 1.18)	0.14	0.56	0.61(0.34, 1.08)	0.09	0.36
Depression severity 1 * Sleep pattern 4	1.74(0.24,12.77)	0.56	0.96	1.76(0.24,12.89)	0.56	0.84
Depression severity 2 * Sleep pattern 1	1.67(0.27,10.23)	0.56	0.96	1.42(0.16, 12.79)	0.68	0.74
Depression severity 2 * Sleep pattern 2	0.80(0.33,1.93)	0.8	0.8	0.67(0.18,2.49)	0.44	0.75
Depression severity 2 * Sleep pattern 3	2.90(1.07,7.88)	0.04	0.24	3.28(1.20,8.96)	0.02	0.12
Depression severity 2 * Sleep pattern 4	1.80(0.21,15.32)	0.57	0.85	2.43(0.31,18.95)	0.38	0.76
Depression severity 3 * Sleep pattern 1	0.60(0.05, 7.58)	0.6	0.8	0.63(0.01, 26.94)	0.63	0.76
Depression severity 3 * Sleep pattern 2	0.52(0.12,2.23)	0.52	1.00	0.46(0.06,3.23)	0.33	0.79
Depression severity 3 * Sleep pattern 3	0.69(0.15, 3.18)	0.61	0.73	0.72(0.19, 2.77)	0.62	0.83
Depression severity 3 * Sleep pattern 4	30.05(0.11,358.54)	0.01	0.12	29.07(3.24,260.38)	0.004	0.048

[Depression severity 1: Moderate depression; Depression severity 2: Moderately severe depression; Depression severity 3: Severe depression; Sleep pattern 1: Poor sleep quality; Sleep pattern 2: Sleep disorders; Sleep pattern 3: Insufficient sleep (≤6h); Sleep pattern 4: Excessive sleep (≥9h)].

(Ref, reference; OR, odds ratio; CI, confidence interval).

*Adjusted P values were corrected via the false discovery rate by using the Benjamin-Hochberg method.

Model1: unadjusted model.

Model2: adjustment for age, sex, race, marital status, education, smoking status, hypertension, diabetes, BMI, PIR.

To further quantify the degree of interaction in relation to asthma risk, we employed measures of RERI, AP, and SI to assess the magnitude of this interaction. We categorized participants into four groups for analysis based on their depressive symptoms severity (moderately severe depression and severe depression) and sleep duration status (insufficient sleep and excessive sleep), as shown in **Tables 6**, **7**. Individuals with both moderately severe depression and insufficient sleep (≤6h) had an increased risk of asthma, with an OR of 3.41 (95%CI, 2.09-5.56, *P <*0.0001) in Model 1, which remained significant after adjusting for covariates factors in Model 2 (OR = 3.10, 95%CI, 1.81-5.29, *P <*0.001). Additionally, Individuals with both severe depression and excessive sleep (≥9h) also exhibited an increased risk of asthma, with an OR of 3.90 (95%CI, 2.44-6.24, *P <*0.0001) in Model 1, which remained significant after adjusting for covariate factors in Model 2 (OR = 3.55, 95%CI, 2.12-5.93, *P <*0.001). In **Table 6**, the results indicated a significant synergy between insufficient sleep (≤6h) and moderately severe depression in relation to asthma in Model 2 (RERI = 1.63, 95%CI = 0.18-3.83; AP = 0.51, 95%CI = 0.15-0.87; SI = 3.92, 95%CI = 1.65-23.50). RERI = 1.63 suggests an additional risk of 1.63 associated with the interaction between insufficient sleep and moderately severe depression in causing asthma, AP = 0.51 suggests 51% of asthma cases in this study sample can be attributed to the interaction between insufficient sleep and moderately severe depression, and SI = 3.92 suggests a significant increase in asthma risk due to the combined effect of these two factors. However, there was no significant synergy between excessive sleep (≥9h) and severe depression in relation to asthma in Model 2 (RERI = 1.74, 95%CI = 0.05-3.54; AP = 0.50, 95%CI = 0.16-0.84; SI = 3.29, 95%CI = 0.84-12.95), as present in **Table 7**.

**Table 6:** Interactive effect analysis of moderately severe depression and insufficient sleep.

Moderately severe depression	Insufficient sleep	Model 1 OR(95% CI)	P value	Model 2 OR(95% CI)	P value
No	No	Ref		Ref	
Yes	No	0.79(0.30,2.07)	0.62	0.97(0.42,1.98)	0.38
No	Yes	1.49(1.23, 1.80)	<0.001	1.42(1.15, 1.76)	0.004
Yes	Yes	3.41(2.09,5.56)	<0.0001	3.10(1.81,5.29)	<0.001
RERI(95%CI)		1.82(0.18, 3.83)		1.63(0.09,3.35)	
AP(95%CI)		0.48(0.11,0.86)		0.51(0.15,0.87)	
SI(95%CI)		2.91(1.77,11.05)		3.92(1.65,23.50)	

(Ref, reference; OR, odds ratio; CI, confidence interval; RERI, relative excess risk of interaction; AP, attribution proportion of interaction; SI, synergy index).

Model1: unadjusted model.

Model2: adjustment for age, sex, race, marital status, education, smoking status, hypertension, diabetes, BMI, PIR.

**Table 7:** Interactive effect analysis of severe depression and excessive sleep.

Severe depression	Excessive sleep	Model 1 OR (95% CI)	P value	Model 2 OR(95% CI)	P value
No	No	Ref		Ref	
Yes	No	0.84(0.38,1.83)	0.64	0.71(0.32,1.59)	0.37
No	Yes	1.47(1.22, 1.78)	<0.001	1.43(1.16, 1.75)	0.003
Yes	Yes	3.90(2.44,6.24)	<0.0001	3.55(2.12,5. 93)	<0.001
RERI(95%CI)		1.88(0.17, 3.93)		1.74(0.05,3.54)	
AP(95%CI)		0.46(0.10,0.82)		0.50(0.16,0.84)	
SI(95%CI)		2.60(0.85,7.92)		3.29(0.84,12.95)	

(Ref, reference; OR, odds ratio; CI, confidence interval; RERI, relative excess risk of interaction; AP, attribution proportion of interaction; SI, synergy index).

Model1: unadjusted model.

Model2: adjustment for age, sex, race, marital status, education, smoking status, hypertension, diabetes, BMI, PIR.

## Discussion

In this study, we conducted a comprehensive analysis of the associations between sleep status, depression, and asthma by utilizing NHANES 2005-2008 data from a cohort of 8,327 individuals. This study is the first to investigate the potential relationship between depression (including its severity) and sleep status (sleep duration, sleep quality, and sleep disorders) on asthma within a large-scale, nationally representative sample. We observed a correlation between depression, poor sleep quality, sleep disorders, insufficient sleep, and the severity of depressive symptoms on asthma even after adjusting for relevant confounding variables. The results of the subgroup analyses and interaction testing indicated that there was effect modification between depression and PIR status, smoking status, and sleep disorders in relation to asthma. We also observed that insufficient sleep had a strengthening effect between moderately severe depression and increased risk of asthma.

Asthma is one of the most common chronic non-communicable diseases worldwide. Individuals with asthma may experience with depression and sleep problems, which can contribute to a higher asthma prevalence. Asthma control is complicated because it is influenced by various factors, including psychological and environmental aspects, as well as comorbidities (33–36). The presence of common asthma comorbidities such as allergic rhinitis, obesity, sleep disorders, depression, and anxiety may increase inflammation, affect the airways, complicate disease management, and increase the risk of treatment failure (34, 36). Therefore, the treatment and management of asthma should consider multiple factors to address the needs of complex case.

Depression is more common in adults with asthma than in the general population. Several studies reported the association between depression and asthma in adults. Previous research has reported that depression might contribute to the onset of asthma and elevated levels of depression in patients with asthma (37, 38). Our study observed a positive association between depression and asthma. Moreover, the relationship between depression and asthma is bidirectional (8, 9). It has been suggested that there may be a shared pathophysiological pathway between depression and asthma, which may be mediated by genetic susceptibility and early exposure to environmental stressors (39). Furthermore, individuals with depression have significantly higher levels of inflammatory cytokines (40), such as interleukin-1 (IL-1), interleukin-4 (IL-4), interleukin-6 (IL-6), and tumor necrosis factor-alpha (TNF-α), which are also important mediators in the pathogenesis of asthma (41). A cohort study founded that severe asthma is associated with higher levels of depression, and inflammatory responses may underlie this comorbid condition (42). Our study found that more severe depressive symptoms exhibited a higher risk of asthma. Additionally, suffering from depression increase the risk of poor adherence to preventive medications and lifestyle changes, affects sleep, and also increases health care utilization, which can further worsen asthma control (43, 44).

Sleep plays a crucial role in maintaining overall health, as the human body undergoes various biological and physiological activities during sleep (45). Asthmatic patients always have highly prevalent of poor sleep quality and sleep disturbance. The detrimental effects of poorly sleep quality on asthma controlled have long been acknowledged (15). Additionally, Sleep disturbance and short sleep duration can lead to systemic low-grade inflammation, which may potentially effect the development of asthma (46). In our study, we observed that participants with asthma exhibited higher PSQI scores, shorter sleep durations, poorer sleep quality, and sleep disorders compared to those without asthma. Individuals with insufficient sleep, poor sleep quality, and sleep disorders had a higher risk of asthma compared to those without sleep disorders. This is consistent with recent studies (19, 47).

Sleep disturbance could manifest as a standalone disease, but it generally coexists with physical or mental diseases, such as depression (48). One study found the mediating effect of sleep quality between dietary behavior and depression symptoms (49). This implies that poor sleep can trigger depression symptoms, and other factors may indirectly affect depression symptoms through their impact on sleep quality. Previous studies identified an interaction between sleep disturbance and depression, leading to an increased risk of various diseases, including cardiovascular, type 2 diabetes mellitus (T2DM), and Parkinson’s disease (50–53). However, research on the interaction between depression and sleep disturbance on asthma is lacking. A previous study found the strong impact of insomnia, anxiety, and depression on asthma-related quality of life (54). A recent study also revealed that depression and sleep disturbance were both related to low health-related quality of life in patients with asthma (55). In addition, another study found a significant increase in the presence of depression among asthmatics with insomnia compared to those without sleep disorders (56).

The relationship between asthma, depression, and sleep problems is complex and may involve biological, psychological, and social modulating factors (44). Our study found that insufficient sleep had a strengthening effect between moderately severe depression and increased risk of asthma. The underlying mechanism in these interactions might involve inflammatory factors. Systemic inflammation may activate microglial cells and astrocytes in brain regions involved in sleep and circadian regulation. These activated cells may secrete pro-inflammatory cytokines (such as IL-1β, TNF-α), nitric oxide, and glial transmitters, which can affect the regulation of sleep homeostasis (57). Sleep deprivation, in turn, can weaken the immune system, leading to increased inflammation (58). This can create a vicious cycle that worsens depression and asthma. There is evidence that psychological therapies or neurological rehabilitation reduce inflammation by modulating the activity of neurocircuitry and the function of brain centers involved in asthma (33).

Family-level factors also play an important role in asthma, especially in children. One study found that physical abuse was associated with poor sleep quality in men at 21 years, while asthma at age 21 showed a modest association with emotional abuse (59). Family function acts as a pathway linking depression and asthma. A previous study observed family chaos as a mechanism underlying the relationship between parental depression and child asthma control (60).

It’s necessary that psychological and sleep assessment be included in asthma management. Additionally, in clinical practice, it is necessary to pay more attention to depression and sleep problems in asthma patients, and to implement intervening for alleviating depression and improving sleep to improve their quality of life. Further longitudinal research is needed to better understand the pathophysiologic mechanisms behind the interactions between asthma, depression, and sleep problems. Cohort studies are also needed to explore whether intervening in depression and improving sleep in asthma patients (with depression and sleep problems) can result in substantial improvements in symptoms and quality of life.

Our study uses the data from the NHANES about Americans is representative, and considered potential covariates that strengthens the generalizability and reliability. This study explored the association between depression, the severity of depressive symptoms, sleep quality, sleep duration, sleep disorders and asthma, and we first researched the interaction between depression and sleep status on asthma. There are several limitations to this study that should be considered. First, the cross-sectional nature of the study does not allow for determination of a causal or bidirectional relationship between depression, sleep status, and asthma. Second, the assessment of sleep was based on self-report, which may be subject to recall and detection bias. Objective measures of sleep, such as polysomnography, would have provided more accurate information. Third, although we used statistical techniques to adjust for confounding variables, the potential confounding factors (such as physical activity levels, dietary habits, medication adherence, and environmental factors) interfering cannot be completely ruled out.

## Conclusion

Our study revealed distinct associations between depression, the severity of depressive symptoms, poor sleep quality, sleep disorders, and insufficient sleep with asthma. Additionally, there was an interaction between moderately severe depression and insufficient sleep on asthma. Psychological and sleep assessment are essential in asthma management. Clinicians should consider the potential risk of depression and sleep problems in asthma patients and intervene. Further longitudinal research is needed to better understand the pathophysiological mechanisms behind the interactions between asthma, depression, and sleep problems.

## Data Availability

The raw data supporting the conclusions of this article will be made available by the authors, without undue reservation.
